# Post-Antibiotic and Post-Antibiotic Sub-Minimum Inhibitory Concentration Effects of Carvacrol against *Salmonella* Typhimurium

**DOI:** 10.3390/ani14182631

**Published:** 2024-09-11

**Authors:** Eva Boyer, Ángela Galán-Relaño, Antonio Romero-Salmoral, Paula Barraza, Lidia Gómez-Gascón, Carmen Tarradas, Inmaculada Luque, Fabiana Carolina de Aguiar, Belén Huerta Lorenzo

**Affiliations:** 1 Animal Health Department, Veterinary Faculty, University of Cordoba, 14014 Cordoba, Spain; ebobuvet@gmail.com (E.B.); antromsal98@gmail.com (A.R.-S.); paula2000bm@gmail.com (P.B.); v32gogal@uco.es (L.G.-G.); sa1taigc@uco.es (C.T.); sa1lumoi@uco.es (I.L.); sa2hulob@uco.es (B.H.L.); 2 Zoonotic and Emerging Diseases (ENZOEM), University of Cordoba, 14014 Cordoba, Spain; 3 Seara Foods, Av. Paludo, 155-Industrial, Seara 89770-000, SC, Brazil; fabianac.aguiar@yahoo.com.br

**Keywords:** minimum inhibitory concentration, post-antibiotic effect, post-antibiotic sub-minimum inhibitory concentration effect, bacterial growth suppression, natural antimicrobials

## Abstract

**Simple Summary:**

Carvacrol, an essential oil compound, has been shown to exhibit antimicrobial activity against pathogens. The aim of this study was to evaluate the post-antibiotic effects (PAE) and post-antibiotic sub-minimum inhibitory concentration (PA-SME) effects of carvacrol on *Salmonella* Typhimurium at different concentrations (1×, 2×, 4× MIC) and inoculum sizes (10^6^ and 10^8^ CFU/mL). The minimum inhibitory concentration (MIC) of carvacrol was established at 0.6 mg/mL, with a required exposure time of 10 min for bacterial inhibition. The results demonstrated that carvacrol exhibited no PAE at the MIC. However, at the 2× MIC, the PAE was observed to be 2 h for the standard inoculum and 1 h for the high-density inoculum. At 4× MIC, the ECP exceeded 43.5 h for both inocula. Further exposure to sub-MIC carvacrol (0.15 mg/mL) after the post-antibiotic phase resulted in an extended ECP of over 43.5 h. These findings suggest that elevated carvacrol sub-MICs can markedly prolong PA-SME, potentially enabling reduced dosing frequencies, minimised adverse effects and enhanced efficacy in the treatment of infected animals and disinfection of agri-food facilities.

**Abstract:**

Carvacrol is a compound present in essential oils with proven antimicrobial activity against numerous pathogens. We firstly determine the post-antibiotic effect (PAE) of carvacrol (1×, 2×, 4× MIC) and post-antibiotic sub-minimum inhibitory concentration (MIC) effect (1× + 0.25× MIC and 2× + 0.25× MIC) for two concentrations of *Salmonella* Typhimurium ATCC14028 (10^6^ and 10^8^ CFU/mL). Prior to testing, the minimum concentration and exposure time to achieve the bacterial inhibition (MIC 0.6 mg/mL and 10 min) were determined by broth microdilution and time–kill curve methods, respectively. At the MIC, carvacrol did not generate any PAE. At twice the MIC, the PAE was 2 h with the standard inoculum (10^6^ CFU/mL) and 1 h with the high-density inoculum (10^8^ CFU/mL). At 4× MIC concentrations, the PAE was higher in both cases > 43.5 h. Continuous exposure of post-antibiotic phase bacteria (1× and 2× MIC) to carvacrol at 0.25× MIC (0.15 mg/mL) resulted in an increase in PAE (PA-SME) above 43.5 h with both inocula. These results suggest that the PA-SME of carvacrol for *S*. Typhimurium can be significantly prolonged by increasing the sub-MICs, which would allow dose spacing, reduce adverse effects and improve its efficacy in the treatment of infected animals and as a disinfectant in agri-food facilities.

## 1. Introduction

Essential oils and their active ingredients, biosynthesised by aromatic plants, are chemical substances authorised for use as disinfectants and food additives (Generally Recognised As Safe, GRAS) [1]. Several studies have demonstrated their antimicrobial potential against a wide range of microorganisms of interest in animal health, as *Salmonella* Typhimurium, which is the second most reported food borne pathogen at the European Union [2]. The antibacterial activity of essential oils has garnered increasing interest in recent years and shown to be effective even against multidrug-resistant isolates [3,4]. The mechanism of action of essential oils is complex, as several components are involved, but the characteristics of these oils can vary depending on the origin; for this reason, there is a trend to analyse the main active principles and their combined effects [5,6].

Carvacrol (C_10_H_14_O) is a liquid phenolic monoterpenoid, 2-methyl-5-(1-methylethyl) phenol, present in the essential oil (EO) of oregano (*Origanum vulgare*), thyme (*Thymus vulgaris*), pepperwort (*Lepidium flavum*), wild bergamot (*Citrus aurantium var. bergamia Loisel*), and other plants [7]. Carvacrol shows minimum inhibitory concentration (MIC) values, understood as the lowest concentration of product capable of inhibiting visible growth of the inoculum, of 194 mg/L, with an interquartile range of 26–780 mg/L against *Salmonella* spp. [8]. However, these parameters, commonly used to determine the in vitro susceptibility of microorganisms, do not provide information on the most appropriate dosage schedule [9].

The PAE refers to the ability of an antimicrobial to inhibit the growth of a bacterium once its serum concentration falls below the MIC. Its most important application is the ability to space out doses, reducing side effects and costs of therapy [10], and it can be determined by the pathogen and the infective dose, the antimicrobial agent, the duration of exposition and the drug concentration [11]. The success of the treatment will also depend on the host immune response and the effect of sub-inhibitory concentrations of the drug prior to its elimination. The PAE is often prolonged if after a short exposure to supra-inhibitory concentrations, the bacteria are re-exposed to sub-MIC concentrations, a process referred to as the PA-SME [9]. Both parameters can be affected by antimicrobial concentration and infecting bacterial dose [12,13].

There are reports on the PAE of conventional antimicrobials against *Salmonella* spp.; however, data on EOs or their active ingredients are very limited. In the present study, we determined, for the first time, the PAE and PA-SME of different concentrations of carvacrol against two different doses of the reference strain *Salmonella* Typhimurium ATCC14028: a standard inoculum (≈10^6^ CFU/mL), equivalent to the minimum infective dose in subclinical carrier animals, and a high-density inoculum (≈10^8^ CFU/mL), equivalent to the infective dose associated with clinical pictures [14].

## 2. Materials and Methods

### 2.1. Bacterial Strain and Tested Product

The *Salmonella* Typhimurium strain (ATCC14028) was obtained from the American Type Culture Collection (ATCC, Manassas, VA, USA), cultured according to the manufacturer’s instructions, and stored at −80 °C in Brain Heart Infusion (BHI) (Oxoid Ltd., Wade Road, Basingstoke, Hampshire, RG24 8PW, UK) supplemented with 20% glycerol. This strain was isolated from the pooled heart and liver tissue of four-week-old chickens and is considered a reference for bioinformatics, enteric disease research, food testing, infectious disease research and pharmaceutical testing (https://www.atcc.org/products/14028, accessed on 1 May 2024).

This strain was tested against different concentrations of carvacrol from Sigma-Aldrich Laboratories (St. Louis, MO, USA) (Natural, 99%, FG).

### 2.2. Determination of MIC

It is crucial to determine the MIC in order to conduct PAE and PA-SME assays. Following the broth microdilution method established by CLSI guidelines [15] for in vitro susceptibility testing with bacteria of animal origin, equal volumes of carvacrol and bacterial inoculum were plated in 96-well microtiter plates. 

From a 16–18 h pure culture, 2–3 colonies were resuspended in sterile saline to an optical density of 0.08–0.1 (measured on an ELISA reader at λ 595 nm), corresponding to a concentration of 1.5 × 10^8^ CFU/mL. From this solution, 100 μL were mixed in 9.9 mL of Müeller-Hinton broth (MHB, Oxoid Ltd., Wade Road, Basingstoke, Hampshire, RG24 8PW, UK) to achieve an inoculum of 1.5 × 10^6^ CFU/mL (final test concentration 5 × 10⁵ CFU/mL).

Carvacrol dilutions were obtained by diluting the compound in MHB with dimethyl sulfoxide (0.1%) in a range from 1.3 to 0.15 mg/mL. To avoid potential pipetting errors, the carvacrol dilutions were prepared in 50 mL tubes and dispensed into the corresponding wells of the 96-well plate. Subsequently, 100 µL of the inoculum and 100 µL of the corresponding dilution of carvacrol were dispensed into each well. To ensure accuracy and reliability, all assays included a positive control (100 μL of broth without antimicrobial and 100 μL of bacterial inoculum) and a negative control (200 μL of broth without antimicrobial and without microorganism). Additionally, quality controls were incorporated, including enrofloxacin and *E. coli* ATCC 25922.

The final bacterial inoculum concentration was determined by mixing 10 μL from the positive control well with 9.9 mL of MHB and then plated 100 μL on three Tryptic Soy Agar plates (TSA, Oxoid Ltd., Wade Road, Basingstoke, Hampshire, RG24 8PW, UK). All plates were incubated at 35 ± 2 °C in aerobiosis for 18 h. The assay was performed in triplicate on different days, and the mean of the results was taken as the final value.

### 2.3. Time–Kill Curve Assay

To determine the minimum exposure time to carvacrol in the PAE and PAE-SME assays, the 24-hours’ time–kill curve study was performed according to the methodology described in document M26-A (CLSI, 2023) [15], using now two bacterial inocula with different concentrations (≈10^6^ and ≈10^8^ CFU/mL). 

Each inoculum was exposed to 0× (growth control), 1×, 2× and 4× carvacrol MIC during 24 h in a water shaking bath (100 rpm) at 35 ± 2 °C. Bacterial growth was monitored by plating serial dilutions onto Mueller–Hinton (MH) plates at times 0, 10 min, and 1, 2, 4, 6, 8 and 24 h. The detection limit of the test was 10 CFU/mL and only plates with 30 to 300 colonies were counted [16]. Each experiment was conducted in triplicate and the average value taken as result. 

The concentration of viable bacteria (log_10_ CFU/mL) per exposure time at the different test concentrations was represented on a semi-log scale using the Microsoft Excel 18.0 software (Microsoft Office 365, Redmond, WA, USA). The antibacterial activity (*E*) was estimated as the difference between the viable bacterial count (log_10_ CFU/mL) at the end (n_t-24_) and at the start (n_t-0_) of the test. Following the criteria of Sidhu et al. [17], three theoretical cut-off points were established: (a) bacteriostatic effect: *E* = 0; there are no changes in value of n_t-0_; (b) bactericidal effect: *E* = −3; there is a reduction of ≥3 log_10_ (99.90%) with respect to the log_10_ of n_t-0_ and (c) virtual eradication of bacteria: *E* = −4; there is a reduction of ≥4 log_10_ (99.99%) with respect to log_10_ of n_t-0_.

The relationship between the carvacrol concentrations and the respective *E* values observed at the end of the assay (24 h) was determined, and the efficiency of each concentration per time was estimated as the reduction percentage of the viable bacteria count with respect to the initial inoculum [16].

### 2.4. PAE and PA-SME Assays

The test was conducted in accordance with the methodology described by Odenholt-Tornqvist et al. [9] and Nedbalcova et al. [12], with adjustments made to the concentrations and exposure time to ensure optimal efficacy according to published results of lethality tests and toxicity profile of carvacrol [18,19,20].

The two bacterial cultures of the strain in logarithmic growth phase were again prepared to a concentration of ≈10^6^ CFU/mL (standard-density inoculum) and ≈10^8^ CFU/mL (high-density inoculum). For each inoculum, two sets (A and B) of 4 tubes were prepared for exposure for 10 min to different concentrations of carvacrol (1×, 2× and 4× MIC) in a water bath at 35 ± 2 °C with agitation (100 rpm). Unexposed bacteria were evaluated in parallel as growth control (carvacrol 0×).

After the initial incubation, the carvacrol was removed by sedimentation of the cells through centrifugation at 3500× *g* for 10 min. The cells were then washed with fresh cation adjusted MHB (without carvacrol) that had been pre-warmed to 35 °C. The process was repeated twice, after which the cells were resuspended in fresh broth (without carvacrol) at 35 °C and incubated at 37 °C with shaking for up to 24 h.

Viable counts were determined by serial dilution and plating on duplicate on MH plates before exposure (time −0.16 h), immediately after the removal of carvacrol (time 0) and after 1, 2, 3, 4, 5, 6, 8, 24, 30 and 48 h of incubation. The control organisms received an identical treatment.

To study the PA-SME of carvacrol, after removal of the product, the second set of tubes (B) was continuously exposed at a sub-MIC concentration of carvacrol (0.25× MIC). One tube with PA-phase bacteria to which no drug was added served as the control. All samples and controls were incubated in a water bath at 35 ± 2 °C with agitation (100 rpm). 

The viable counts (log_10_ CFU/mL) of PAE were plotted vs. time with the software Microsoft Excel (Microsoft Office 365, USA). The PAE was defined as PAE = T − C, where T is the time required for the viable count in the test culture to increase 1 log_10_ over the count observed at time zero, and C represents the corresponding time for the carvacrol-free control. The PA-SME was defined according to the formula PA-SME = T_PA_ − C, where T_PA_ is the time for the cultures previously exposed to carvacrol, which thereafter had been exposed to sub-MIC, to increase 1 log_10_ unit above the counts observed immediately after washing, and C is the corresponding time for the unexposed control [21]. The PAE and PA-SME were measured in three independent experiments and the average value taken as result. The Student’s *t* test was used to determine the significant differences (*p* < 0.05) between carvacrol tested concentrations and bacterial inoculum densities [21].

## 3. Results

### 3.1. MIC Assay Results

The mean MIC value of *S*. Typhimurium ATCC 14028 against carvacrol was 0.6 mg/mL.

### 3.2. Time–Kill Curve Results

**Figure 1:**
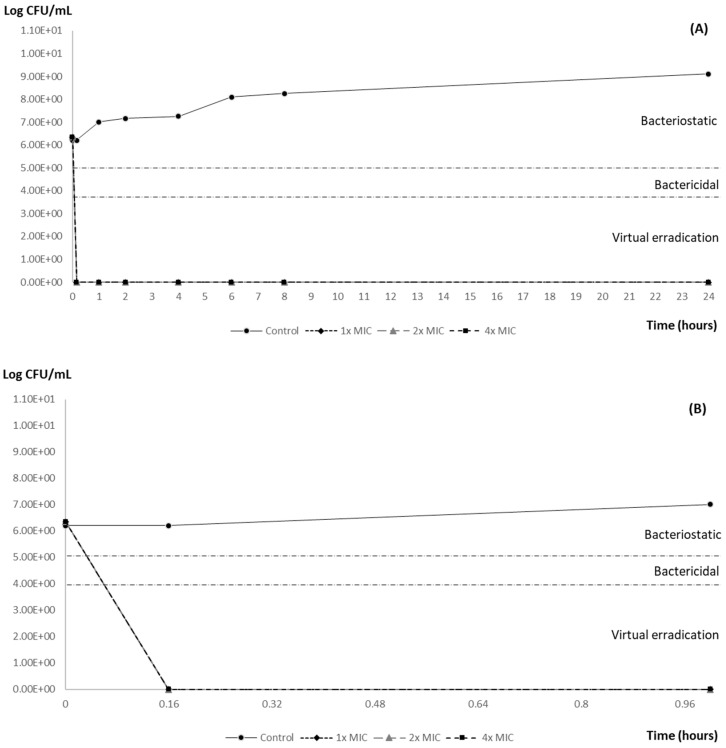
(**A**) Lethality curve of different concentrations of carvacrol against a 10^6^ CFU/mL inoculum of *Salmonella* Typhimurium ATCC 14028. (**B**) Enlargement of the results obtained in the first hour of the test. The horizontal dotted lines represent the theoretical cut-off points to evaluate the efficacy of the antimicrobial (reduction with respect to the initial inoculum): bacteriostatic effect (reduction ≥ 2 log_10_), bactericidal (reduction ≥ 3 log_10_) and virtual eradication of bacteria (reduction ≥ 4 log_10_). MIC 600 μg/mL.

**Figure 2:**
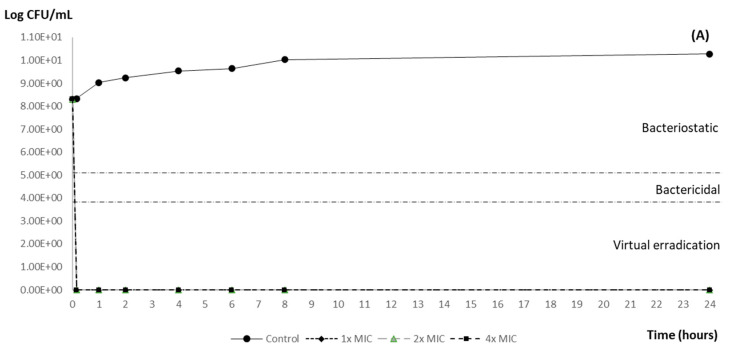

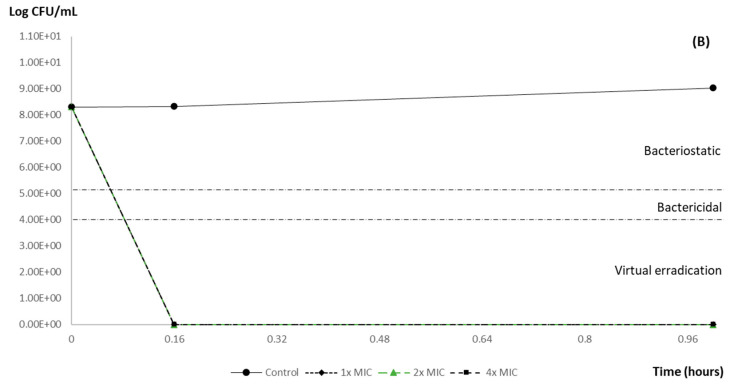
(**A**) Lethality curve of different concentrations of carvacrol against a 10^8^ CFU/mL inoculum of *Salmonella* Typhimurium ATCC 14028. (**B**) Enlargement of the results obtained in the first hour of the test. The horizontal dotted lines represent the theoretical cut-off points to evaluate the efficacy of the antimicrobial (reduction with respect to the initial inoculum): bacteriostatic effect (reduction ≥ 2 log_10_), bactericidal (reduction ≥ 3 log_10_) and virtual eradication of bacteria (reduction ≥ 4 log_10_). MIC 600 μg/mL.

Carvacrol showed concentration-dependent antimicrobial activity against the tested strain (Figure 1 and Figure 2). All the carvacrol concentrations (1×, 2× and 4× MIC: 0.6, 1.2 and 2.4 mg/mL, respectively) achieved 100% elimination (virtual eradication) of both inocula within ten minutes of exposure (Table 1). Efficacy was maintained in all cases until the end of the assay.

### 3.3. PAE and PA-SME Assays Results

**Figure 3:**
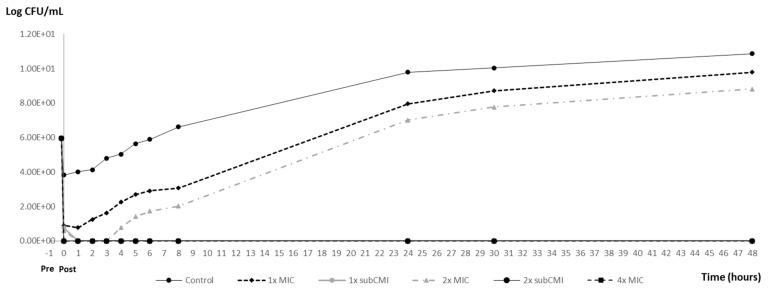
PAE and PA-SME of different carvacrol concentrations for a standard-density inoculum (10^6^ CFU/mL) of *S*. Typhimurium ATCC 14028. Pre: The time of beginning exposure to the carvacrol. Post: The time of discontinuing exposure to the carvacrol. MIC = 600 μg/mL; subMIC tested = 150 μg/mL.

**Figure 4:**
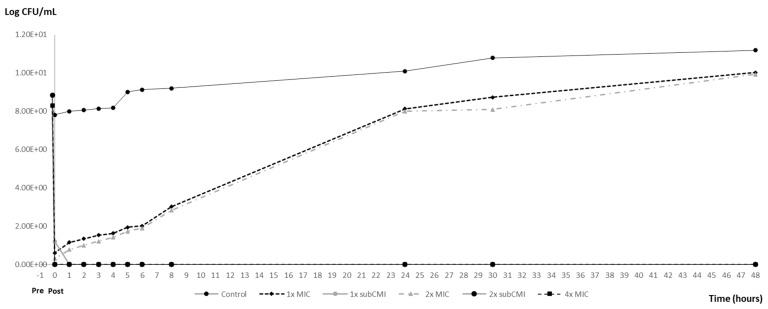
PAE and PA-SME of different carvacrol concentrations for a high-density inoculum (10^8^ CFU/mL) of *S*. Typhimurium ATCC 14028. Pre: The time of beginning exposure to the carvacrol. Post: The time of discontinuing exposure to the carvacrol. MIC = 600 μg/mL; subMIC tested = 150 μg/mL.

Based on the time–kill curve, PAE and PA-SME were assessed for an initial carvacrol exposure of 10 min. Table 2 shows the PAE and PA-SME (0.25×) effects observed for the standard inoculum (10^6^ CFU/mL) and the high-density inoculum (10^8^ CFU/mL) of *S*. Typhimurium ATCC 14028. Figure 3 and Figure 4 illustrate the variations in bacterial counts over time for the standard and the high-density inocula, respectively.

As the graphs demonstrate, the PAE was only observed after the exposure of the inocula to supra-inhibitory concentrations of carvacrol. In particular, exposure to a 2× MIC concentration (1.2 mg/mL) was found to inhibit the growth of inoculum 10^6^ CFU/mL for 2 h and of inoculum 10^8^ CFU/mL for 1 h (*p* < 0.05). Following this period, bacterial growth reactivated exponentially, reaching values close to the positive control in both cases. However, exposure to a 4× MIC concentration (2.4 mg/mL) resulted in a significant increase in the PAE (*p* < 0.05), exceeding 44.5 h with the standard inoculum and 43.5 h with the high-density inoculum.

Further exposure of bacteria that had previously been exposed to antibiotics (1× and 2× MIC) to carvacrol at 0.25× MIC (0.15 mg/mL) resulted in an increased PA-SME over 44.5 h for the standard inoculum and 43.5 h for the high-density inoculum (Figure 2 and Figure 3), which represents a notable divergence from the PAE observed with these same concentrations.

## 4. Discussion

Pharmacodynamics allows us to evaluate the therapeutic efficacy of antimicrobials based on the plasma concentration they reach and the MIC of each microorganism. There are concentration-dependent antimicrobials (their action is related to the plasma concentration) and time-dependent antimicrobials (their action is related to the time they are present in concentrations over the MIC). In general, concentration-dependent drugs (e.g., aminoglycosides, fluoroquinolones) have a longer PAE, which allows for dose spacing (single daily dose in the case of aminoglycosides) and reduced toxicity. In contrast, time-dependent antimicrobials (e.g., beta-lactams) have a shorter PAE, making continuous administration a more convenient option [22].

Despite the evident utility of the data yielded by PAE and other parameters, and the concurrent necessity to identify alternative antimicrobial agents for disease control, the available literature lacks pertinent information. This study represents the first evaluation of the PAE in natural antimicrobials, with the results demonstrating that carvacrol exhibits concentration-dependent bactericidal activity against *Salmonella* Typhimurium and a notable PAE at supra-inhibitory doses. In terms of bacterial-killing kinetics, the findings differ significantly from those reported by Guimarães et al. [23] using the same product and strain. These authors state that carvacrol exhibits inhibitory activity at a MIC of 0.015 mg/mL (test range 0.002 to 0.25 mg/mL) and no bactericidal activity. In contrast, our assay yielded an estimated MIC of 0.6 mg/mL (assay range 0.15–1.3 mg/mL), accompanied by a 100% reduction in the test inoculum after 10 min of exposure. Our findings align with those previously reported by De Aguiar et al. [24] for *Streptococcus suis*, demonstrating complete eradication of the bacterium after 5 min of exposure to doses of carvacrol at 2× and 4× MIC, 0.3125 and 0.625 mg/mL, respectively.

With regard to the PAE exhibited by carvacrol for the inoculum of 10^6^ CFU/mL, which is equivalent to the minimum dose associated with subclinical infection in immunocompetent animals [14], the period was 2 h after a 10 min exposure to 2× MIC, and more than 44.5 h after exposure to 4× MIC.

Both effects are significantly higher than those described by Wain et al. [25] for *Salmonella* Typhi strains after exposure to orbifloxacin (4× MIC/30 min; PAE = 3 h, range 1–4 h) and ceftriaxone (4× MIC/1 h; PAE = 0 h). Other studies showed a PAE of orbifloxacin and enrofloxacin against *E. coli* isolates after 1 h exposure to at 2× MIC of 0.29 h and 0.32 h, respectively [21].

The effect of the antimicrobial concentration on PAE has also been reported in previous studies with respiratory pathogens and veterinary antibiotics, such as amoxicillin, marbofloxacin and enrofloxacin [12]. In this work, exposure for 1 h to 5× MIC antibiotic concentrations induced a PAE for *Actinobacillus pleuropneumoniae* of 8.3 h (6–10 h) with fluoroquinolones and 4 h (2–6 h) with beta-lactams. By increasing the antibiotic concentration to 10× MIC, a significant increase in PAE was detected up to 9.0 h (8–12 h) with marbofloxacin, 8.6 h (8–10 h) with enrofloxacin and 5.7 h (3–6 h) with amoxicillin, respectively. Furthermore, differences in PAE depending on the microorganism were observed. In the case of *Pasteurella multocida*, the PAE of marbofloxacin against *P. multocida* was of 5.1 h (2–6 h), whereas with enrofloxacin was of 3.5 h (2–6 h) and 1.3 h (1–4 h) when amoxicillin was used. At 10× MIC concentrations, the PAE of the fluoroquinolones increased significantly to 5.6 h (3–6 h) and 4.4 h (3–6 h). In the case of amoxicillin, no changes were observed. These results are significantly lower than those obtained in our study with carvacrol at 4× MIC (PAE ≥ 44.5 h).

In our study, the effect of inoculum size on the PAE was evaluated, for that, the assays were carried out with an inoculum. In this case, at a concentration of carvacrol 1× and 2×, PAE was of 0 h and 1 h, respectively, in agreement with those obtained for rifampicin 5× MIC (PAE 0.88 h) and ceftriaxone 10× MIC (PAE 0 h) [26]. However, in our work, carvacrol at 4× MIC showed a PAE ≥ 43.5 h. 

The chemo-computational toxicity prediction, performed by Akermi et al. [27], with Protox II webserver, showed that carvacrol could be safely and effectively used as drug candidates to tackle bacterial, fungal, and viral infections. On the other hand, in vivo acute oral toxicity studies in rats showed a lethal dose 50 (LD50) of 800 mg/kg in rats and 100 mg/kg in rabbits [20], both below the threshold of 2000 mg/kg established by the Organisation for Economic Cooperation and Development (OECD, 2008). The genotoxic potential of carvacrol is very weak, although its action on DNA cannot be ruled out due to the nuclear fragmentation observed at concentrations of 10 to 160 mg/L [18,19].

The prolongation of PAE by re-exposure of bacteria to concentrations below the MIC has been evidenced. This phenomenon is designated as the post-antibiotic subminimum inhibitory concentration effect (PA-SME), as previously described. Although the action mechanism is not known, early studies by Yan et al. [28] and Odenholt-Tornqvist et al. [9], with beta-lactams and fluoroquinolones, suggested that the PAE is the time needed for the bacteria to recover altered functions (PBP protein synthesis, protein translocation, etc.) and re-enter a logarithmic growth phase. During this time, only small amounts of antibiotic would be needed to limit the growth of these bacteria (PAE-SME). Also, based on the presence in the bacterial populations of more sensitive subpopulations that are inhibited at sub-MIC concentrations, these authors suggested that the PA-SME would represent the time it takes for the most resistant subpopulation to become dominant. 

Essential oils and their active ingredients are commonly used in animal feed as additives and probiotics and in the food industry for the development of new active packaging systems [29,30]. The EFSA expert evaluation of the safety of a carvacrol-based feed additive in various animal species (broilers, weaned piglets and dairy cows) carried out in 2017 [31] demonstrated that at the recommended use level (150 mg additive/kg feed), it is safe for broilers and weaned piglets. This conclusion extends to all poultry and swine species reared for meat production. Furthermore, doses of 500 mg of the additive per head per day (equivalent to approximately 25 mg per kg of complete feed) have been demonstrated to be safe for the dairy cow. The residue studies (meat, liver, fatty milk and eggs) demonstrate that consumer exposure to products from animals administered the additive at the recommended use level did not give rise to safety concerns. However, contact with the pure additive may cause skin and mucosal irritation and has the potential for sensitisation in susceptible individuals. Moreover, the utilisation of carvacrol in animal husbandry is not anticipated to engender any adverse effects on the surrounding environment. Although bacterial strains may potentially evolve resistance to this compound over time, there is currently a paucity of documented instances of such resistance to carvacrol, which leads it an efficacious alternative for the management of bacterial populations.

In our study, continuous post-antibiotic exposure of both inocula of *S.* Typhimurium to carvacrol at 0.25× MIC (0.15 mg/mL) resulted in an increase in PAE of the 1× and 2× MIC concentrations up to 43.5 h. This increase was much higher than that found by Harada et al. [21] for *E. coli* strains after re-exposure to orbifloxacin and enrofloxacin at 0.1×, 0.2× and 0.3× MIC concentrations (0.55 and 0.58 h, 1.11 and 0.87 h, and 2.03 and 1.38 h, respectively).

## 5. Conclusions

Our study demonstrated for the first time that carvacrol could have an important PAE and PA-SME against *Salmonella* spp., which would support its use as an alternative to traditional antimicrobials in the treatment of infected animals and as a disinfectant in agri-food facilities. However, further research is needed with a larger number of strains and to extend the time to measure the effects beyond 48 h, in order to adjust the application interval.

## Figures and Tables

**Table 1:** Percentage reduction in the number of viable bacteria with respect to the initial inoculum obtained for each concentration of carvacrol and assay time [16].

**Post-Exposition Time**	**Carvacrol Efficacy with Inoculum 10^6^**		
	**1× CMI** **(0.6 mg/mL)**	**2× CMI** **(1.2 mg/mL)**	**4× CMI** **(2.4 mg/mL)**
10 min	100%	100%	100%
1 h	100%	100%	100%
2 h	100%	100%	100%
4 h	100%	100%	100%
8 h	100%	100%	100%
24 h	100%	100%	100%
**Post-Exposition Time**	**Carvacrol Efficacy with Inoculum 10^8^**		
	**1× CMI** **(0.6 mg/mL)**	**2× CMI** **(1.2 mg/mL)**	**4× CMI** **(2.4 mg/mL)**
10 min	100%	100%	100%
1 h	100%	100%	100%
2 h	100%	100%	100%
4 h	100%	100%	100%
8 h	100%	100%	100%
24 h	100%	100%	100%

**Table 2:** Carvacrol post-antibiotic effect (PAE) and post-antibiotic sub-minimum inhibitory concentration effect (PA-SME) against a standard inoculum and a high-density inoculum of *Salmonella* Typhimurium ATCC 14028 strain.

Inoculum CFU/mL	PAEs (h)			PA-SMEs (h)	
	1× MIC (0.6 mg/mL)	2× MIC (1.2 mg/mL)	4× MIC (2.4 mg/mL)	1× + 0.25 MIC (0.6 + 0.15 mg/mL)	2× + 0.25 MIC (1.2 + 0.15 mg/mL)
**10^6^**	0 ± 0	2 ± 0	>44.5 ± 0	>44.5 ± 0	>44.5 ± 0
**10^8^**	0 ± 0	1 ± 0	>43.5 ± 0	>43.5 ± 0	>43.5 ± 0

## Data Availability

Data are contained within the article.
